# Benzyl 2-(4-bromo­anilino)-4,4-dimethyl-6-oxocyclo­hex-1-enecarbodithio­ate: first triclinic polymorph

**DOI:** 10.1107/S160053680900614X

**Published:** 2009-02-25

**Authors:** El Sayed H. El Ashry, Mohammed R. Amer, Muhammad Raza Shah, Seik Weng Ng

**Affiliations:** a H.E.J. Research Institute of Chemistry, International Center for Chemical and Biological Sciences, University of Karachi, Karachi 75270, Pakistan; b Department of Chemistry, University of Malaya, 50603 Kuala Lumpur, Malaysia

## Abstract

The six-membered cyclo­hexene ring in the title compound, C_22_H_22_BrNOS_2_, adopts an envelope conformation, with the C atom bearing the two methyl groups representing the flap. This atom deviates by 0.686 (4) Å from the plane passing through the other five atoms of the ring (r.m.s. deviation = 0.025 Å). The mol­ecular conformation is stabilized by an intra­molecular N—H⋯S hydrogen bond.

## Related literature

For previous work on this topic, see: El Ashry *et al.* (2005*a* ▶,*b* ▶, 2006 ▶, 2008*a* ▶,*b* ▶); El Ashry, Kassem *et al.* (2009 ▶). For the use of enamines in heterocyclic synthesis, see: Tominaga (1989 ▶); Tominaga *et al.* (1991 ▶). For another triclinic polymorph of the title compound, see El Ashry, Amer *et al.* (2009 ▶).
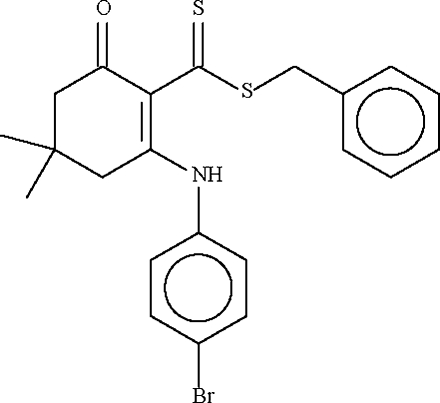


## Experimental

### 

#### Crystal data

C_22_H_22_BrNOS_2_
*M* *_r_* = 460.44
Triclinic, 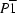

*a* = 9.3030 (3) Å
*b* = 9.8099 (3) Å
*c* = 12.3538 (4) Å
α = 73.464 (2)°
β = 72.391 (2)°
γ = 89.916 (2)°
*V* = 1025.73 (6) Å^3^
*Z* = 2
Mo *K*α radiation
μ = 2.22 mm^−1^
*T* = 100 K
0.40 × 0.04 × 0.04 mm

#### Data collection

Bruker SMART APEX diffractometer
Absorption correction: multi-scan (*SADABS*; Sheldrick, 1996 ▶) *T*_min_ = 0.684, *T*_max_ = 0.917
9757 measured reflections
4709 independent reflections
3490 reflections with *I* > 2σ(*I*)
*R*_int_ = 0.039

#### Refinement

*R*[*F* ^2^ > 2σ(*F* ^2^)] = 0.037
*wR*(*F* ^2^) = 0.077
*S* = 0.99
4709 reflections
250 parameters
1 restraint
H atoms treated by a mixture of independent and constrained refinement
Δρ_max_ = 0.47 e Å^−3^
Δρ_min_ = −0.38 e Å^−3^

### 

Data collection: *APEX2* (Bruker, 2008 ▶); cell refinement: *SAINT* (Bruker, 2008 ▶); data reduction: *SAINT*; program(s) used to solve structure: *SHELXS97* (Sheldrick, 2008 ▶); program(s) used to refine structure: *SHELXL97* (Sheldrick, 2008 ▶); molecular graphics: *X-SEED* (Barbour, 2001 ▶); software used to prepare material for publication: *publCIF* (Westrip, 2009 ▶).

## Supplementary Material

Crystal structure: contains datablocks global, I. DOI: 10.1107/S160053680900614X/bt2876sup1.cif

Structure factors: contains datablocks I. DOI: 10.1107/S160053680900614X/bt2876Isup2.hkl

Additional supplementary materials:  crystallographic information; 3D view; checkCIF report

## Figures and Tables

**Table 1:** Hydrogen-bond geometry (Å, °)

*D*—H⋯*A*	*D*—H	H⋯*A*	*D*⋯*A*	*D*—H⋯*A*
N1—H1⋯S2	0.88 (1)	2.10 (2)	2.905 (2)	151 (3)

**Fig. 1.**
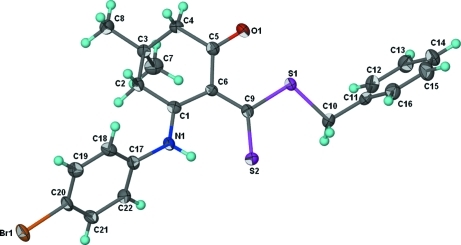
Anisotropic displacement ellipsoid plot (Barbour, 2001) plot of C22H22BrNOS2 at the 70% probability level; hydrogen atoms are drawn as spheres of arbitrary radius.

## supplementary crystallographic information

### Comment

The enaminodithiocarboxylates are a class of compounds of crucial significance for designing functionalized molecules that are suitable for studying the biological activity of potentially useful chemicals. They are also important reactants in the synthesis of heterocyclic compounds (Tominaga, 1989; Tominaga *et al.*, 1991). Our interest in such class of compounds revolves around new glycosyl donors (El Ashry *et al.*, 2005*a*, 2005*b*, 2006, 2008*a*, 2008*b*, El Ashry, Kassem *et al.*2009). The title compound is to be used in a model study to understand the scope of the reaction that uses cyclic enaminones. A possibility exists that the representative title compound can lead to a fused cyclized product.

### Experimental

To a solution of 3-(4-bromoanilino)-5,5-dimethyl-cyclohex-2-en-1-one (0.1 mol) in DMSO (20 ml) and sodium hydroxide (0.1 mol) in water (1 ml) was added carbon disulphide (0.3 mol). The mixture was kept at 263 K for 20 min. Benzyl bromide (0.1 mol) was added. The mixture was left for 24 h, after which it was quenched with water (200 ml) and then acidified with 10% hydrochloric acid. The resulting precipitate was collected by filtration, dried and purified on a silica gel column (30% ethyl acetate in hexane) to give orange crystals (40% yield, mp 458 K). HRMS for C_22_H_22_BrNOS_2_, calc.: 459.0326, found, *m/z*: 459.0316.

### Refinement

Carbon-bound H-atoms were placed in calculated positions (C—H 0.93 to 0.99 Å) and were included in the refinement in the riding model approximation, with *U*(H) set to 1.2 to 1.5*U*(C). The methyl groups were allowed to rotate but not to tip.

The amino H-atom was located in a difference Fourier map, and was refined with a distance restraint of N–H 0.88±0.01 Å; its isotropic displacement parameter was freely refined.

### Crystal data

**Table:**

C_22_H_22_BrNOS_2_	*Z* = 2
*M**_r_* = 460.44	*F*(000) = 472
Triclinic, *P*1	*D*_x_ = 1.491 Mg m^−^^3^
Hall symbol: -P 1	Mo *K*α radiation, λ = 0.71073 Å
*a* = 9.3030 (3) Å	Cell parameters from 2124 reflections
*b* = 9.8099 (3) Å	θ = 2.4–25.2°
*c* = 12.3538 (4) Å	µ = 2.22 mm^−^^1^
α = 73.464 (2)°	*T* = 100 K
β = 72.391 (2)°	Prism, yellow
γ = 89.916 (2)°	0.40 × 0.04 × 0.04 mm
*V* = 1025.73 (6) Å^3^	

### Data collection

**Table:**

Bruker SMART APEX diffractometer	4709 independent reflections
Radiation source: fine-focus sealed tube	3490 reflections with *I* > 2σ(*I*)
graphite	*R*_int_ = 0.039
ω scans	θ_max_ = 27.5°, θ_min_ = 1.8°
Absorption correction: multi-scan (*SADABS*; Sheldrick, 1996)	*h* = −11→12
*T*_min_ = 0.684, *T*_max_ = 0.917	*k* = −12→12
9757 measured reflections	*l* = −16→15

### Refinement

**Table:**

Refinement on *F*^2^	Primary atom site location: structure-invariant direct methods
Least-squares matrix: full	Secondary atom site location: difference Fourier map
*R*[*F*^2^ > 2σ(*F*^2^)] = 0.037	Hydrogen site location: inferred from neighbouring sites
*wR*(*F*^2^) = 0.077	H atoms treated by a mixture of independent and constrained refinement
*S* = 0.99	*w* = 1/[σ^2^(*F*_o_^2^) + (0.0236*P*)^2^ + 0.5453*P*] where *P* = (*F*_o_^2^ + 2*F*_c_^2^)/3
4709 reflections	(Δ/σ)_max_ = 0.001
250 parameters	Δρ_max_ = 0.47 e Å^−^^3^
1 restraint	Δρ_min_ = −0.37 e Å^−^^3^

### Special details

**Table:**

Geometry. All e.s.d.'s (except the e.s.d. in the dihedral angle between two l.s. planes) are estimated using the full covariance matrix. The cell e.s.d.'s are taken into account individually in the estimation of e.s.d.'s in distances, angles and torsion angles; correlations between e.s.d.'s in cell parameters are only used when they are defined by crystal symmetry. An approximate (isotropic) treatment of cell e.s.d.'s is used for estimating e.s.d.'s involving l.s. planes.

### Fractional atomic coordinates and isotropic or equivalent isotropic displacement parameters (Å^2^)

**Table:**

	*x*	*y*	*z*	*U*_iso_*/*U*_eq_	
Br1	0.36770 (4)	0.20219 (3)	1.10143 (3)	0.02491 (9)	
S1	0.64466 (8)	1.01099 (7)	0.25672 (6)	0.01823 (16)	
S2	0.74397 (8)	0.76685 (8)	0.41055 (6)	0.02190 (17)	
O1	0.3727 (2)	1.0432 (2)	0.34593 (18)	0.0319 (5)	
N1	0.4826 (3)	0.6419 (2)	0.6181 (2)	0.0182 (5)	
H1	0.5775 (16)	0.658 (3)	0.571 (2)	0.035 (10)*	
C1	0.3918 (3)	0.7277 (3)	0.5732 (2)	0.0153 (6)	
C2	0.2258 (3)	0.7000 (3)	0.6438 (2)	0.0181 (6)	
H2A	0.2056	0.7509	0.7048	0.022*	
H2B	0.2024	0.5966	0.6860	0.022*	
C3	0.1189 (3)	0.7459 (3)	0.5701 (2)	0.0179 (6)	
C4	0.1689 (3)	0.9016 (3)	0.5000 (2)	0.0196 (6)	
H4A	0.1096	0.9325	0.4443	0.024*	
H4B	0.1437	0.9594	0.5560	0.024*	
C5	0.3345 (3)	0.9339 (3)	0.4300 (2)	0.0192 (6)	
C6	0.4459 (3)	0.8392 (3)	0.4637 (2)	0.0149 (6)	
C7	0.1277 (3)	0.6549 (3)	0.4872 (3)	0.0267 (7)	
H7A	0.0547	0.6832	0.4439	0.040*	
H7B	0.1040	0.5540	0.5340	0.040*	
H7C	0.2302	0.6689	0.4304	0.040*	
C8	−0.0436 (3)	0.7327 (3)	0.6537 (3)	0.0256 (7)	
H8A	−0.1117	0.7662	0.6067	0.038*	
H8B	−0.0483	0.7909	0.7072	0.038*	
H8C	−0.0747	0.6326	0.7007	0.038*	
C9	0.6016 (3)	0.8658 (3)	0.3878 (2)	0.0158 (6)	
C10	0.8407 (3)	0.9962 (3)	0.1768 (2)	0.0211 (6)	
H10A	0.9090	1.0131	0.2204	0.025*	
H10B	0.8531	0.9004	0.1655	0.025*	
C11	0.8740 (3)	1.1104 (3)	0.0584 (2)	0.0198 (6)	
C12	0.8062 (3)	1.0952 (3)	−0.0244 (3)	0.0243 (7)	
H12	0.7392	1.0127	−0.0055	0.029*	
C13	0.8346 (3)	1.1978 (3)	−0.1334 (3)	0.0290 (7)	
H13	0.7881	1.1855	−0.1891	0.035*	
C14	0.9310 (4)	1.3184 (3)	−0.1609 (3)	0.0306 (8)	
H14	0.9511	1.3891	−0.2358	0.037*	
C15	0.9979 (4)	1.3362 (3)	−0.0802 (3)	0.0306 (8)	
H15	1.0637	1.4194	−0.0992	0.037*	
C16	0.9693 (3)	1.2323 (3)	0.0298 (3)	0.0250 (7)	
H16	1.0156	1.2455	0.0853	0.030*	
C17	0.4444 (3)	0.5381 (3)	0.7324 (2)	0.0168 (6)	
C18	0.3865 (3)	0.5766 (3)	0.8354 (2)	0.0203 (6)	
H18	0.3649	0.6724	0.8308	0.024*	
C19	0.3599 (3)	0.4755 (3)	0.9452 (3)	0.0212 (6)	
H19	0.3188	0.5013	1.0163	0.025*	
C20	0.3937 (3)	0.3364 (3)	0.9507 (2)	0.0197 (6)	
C21	0.4509 (3)	0.2970 (3)	0.8484 (2)	0.0202 (6)	
H21	0.4732	0.2014	0.8531	0.024*	
C22	0.4755 (3)	0.3981 (3)	0.7388 (2)	0.0202 (6)	
H22	0.5137	0.3715	0.6678	0.024*	

### Atomic displacement parameters (Å^2^)

**Table:**

	*U*^11^	*U*^22^	*U*^33^	*U*^12^	*U*^13^	*U*^23^
Br1	0.02959 (18)	0.02257 (16)	0.01791 (15)	−0.00197 (12)	−0.00961 (12)	0.00337 (12)
S1	0.0185 (4)	0.0162 (3)	0.0149 (3)	0.0032 (3)	−0.0018 (3)	−0.0005 (3)
S2	0.0162 (4)	0.0236 (4)	0.0205 (4)	0.0054 (3)	−0.0041 (3)	−0.0002 (3)
O1	0.0258 (12)	0.0285 (12)	0.0251 (12)	0.0089 (10)	−0.0013 (9)	0.0094 (9)
N1	0.0154 (13)	0.0186 (12)	0.0158 (12)	0.0009 (10)	−0.0043 (10)	0.0014 (10)
C1	0.0181 (15)	0.0129 (13)	0.0176 (14)	0.0016 (11)	−0.0075 (12)	−0.0066 (11)
C2	0.0177 (15)	0.0172 (14)	0.0153 (14)	0.0004 (11)	−0.0040 (11)	−0.0001 (11)
C3	0.0163 (15)	0.0191 (14)	0.0175 (14)	0.0018 (12)	−0.0060 (12)	−0.0035 (12)
C4	0.0173 (15)	0.0186 (14)	0.0209 (15)	0.0046 (12)	−0.0054 (12)	−0.0036 (12)
C5	0.0205 (15)	0.0197 (15)	0.0154 (14)	0.0034 (12)	−0.0049 (12)	−0.0027 (12)
C6	0.0153 (14)	0.0166 (13)	0.0126 (13)	0.0035 (11)	−0.0060 (11)	−0.0023 (11)
C7	0.0238 (17)	0.0280 (16)	0.0337 (18)	0.0017 (13)	−0.0137 (14)	−0.0124 (14)
C8	0.0162 (15)	0.0254 (16)	0.0288 (17)	0.0011 (13)	−0.0051 (13)	−0.0004 (13)
C9	0.0185 (14)	0.0148 (13)	0.0134 (13)	0.0004 (11)	−0.0048 (11)	−0.0034 (11)
C10	0.0168 (15)	0.0216 (15)	0.0199 (15)	0.0026 (12)	−0.0010 (12)	−0.0036 (12)
C11	0.0167 (15)	0.0192 (14)	0.0190 (15)	0.0046 (12)	0.0003 (12)	−0.0052 (12)
C12	0.0210 (16)	0.0254 (16)	0.0224 (16)	−0.0034 (13)	−0.0029 (13)	−0.0051 (13)
C13	0.0250 (17)	0.0389 (19)	0.0197 (16)	0.0040 (14)	−0.0079 (13)	−0.0026 (14)
C14	0.0350 (19)	0.0254 (17)	0.0206 (16)	0.0059 (15)	−0.0027 (14)	0.0032 (13)
C15	0.0343 (19)	0.0179 (15)	0.0315 (18)	−0.0040 (14)	−0.0017 (15)	−0.0042 (14)
C16	0.0258 (17)	0.0248 (16)	0.0229 (16)	0.0013 (13)	−0.0054 (13)	−0.0072 (13)
C17	0.0150 (14)	0.0180 (14)	0.0160 (14)	−0.0014 (11)	−0.0068 (11)	−0.0009 (11)
C18	0.0227 (16)	0.0164 (14)	0.0236 (15)	0.0029 (12)	−0.0106 (13)	−0.0054 (12)
C19	0.0236 (16)	0.0234 (15)	0.0189 (15)	0.0034 (13)	−0.0095 (12)	−0.0067 (12)
C20	0.0191 (15)	0.0189 (14)	0.0165 (14)	−0.0018 (12)	−0.0072 (12)	0.0033 (12)
C21	0.0220 (16)	0.0139 (14)	0.0232 (15)	0.0014 (12)	−0.0076 (12)	−0.0026 (12)
C22	0.0207 (15)	0.0221 (15)	0.0151 (14)	0.0040 (12)	−0.0037 (12)	−0.0035 (12)

### Geometric parameters (Å, °)

**Table:**

Br1—C20	1.895 (3)	C8—H8C	0.9800
S1—C9	1.763 (3)	C10—C11	1.511 (4)
S1—C10	1.820 (3)	C10—H10A	0.9900
S2—C9	1.686 (3)	C10—H10B	0.9900
O1—C5	1.224 (3)	C11—C16	1.384 (4)
N1—C1	1.321 (3)	C11—C12	1.394 (4)
N1—C17	1.427 (3)	C12—C13	1.383 (4)
N1—H1	0.884 (10)	C12—H12	0.9500
C1—C6	1.424 (3)	C13—C14	1.382 (4)
C1—C2	1.506 (4)	C13—H13	0.9500
C2—C3	1.527 (4)	C14—C15	1.373 (4)
C2—H2A	0.9900	C14—H14	0.9500
C2—H2B	0.9900	C15—C16	1.396 (4)
C3—C4	1.518 (4)	C15—H15	0.9500
C3—C7	1.524 (4)	C16—H16	0.9500
C3—C8	1.532 (4)	C17—C18	1.382 (4)
C4—C5	1.504 (4)	C17—C22	1.388 (4)
C4—H4A	0.9900	C18—C19	1.385 (4)
C4—H4B	0.9900	C18—H18	0.9500
C5—C6	1.465 (4)	C19—C20	1.388 (4)
C6—C9	1.444 (4)	C19—H19	0.9500
C7—H7A	0.9800	C20—C21	1.379 (4)
C7—H7B	0.9800	C21—C22	1.385 (4)
C7—H7C	0.9800	C21—H21	0.9500
C8—H8A	0.9800	C22—H22	0.9500
C8—H8B	0.9800		
			
C9—S1—C10	104.06 (13)	C6—C9—S1	116.85 (19)
C1—N1—C17	127.3 (2)	S2—C9—S1	117.21 (16)
C1—N1—H1	113 (2)	C11—C10—S1	104.63 (18)
C17—N1—H1	120 (2)	C11—C10—H10A	110.8
N1—C1—C6	122.4 (2)	S1—C10—H10A	110.8
N1—C1—C2	116.2 (2)	C11—C10—H10B	110.8
C6—C1—C2	121.4 (2)	S1—C10—H10B	110.8
C1—C2—C3	114.7 (2)	H10A—C10—H10B	108.9
C1—C2—H2A	108.6	C16—C11—C12	118.4 (3)
C3—C2—H2A	108.6	C16—C11—C10	121.7 (3)
C1—C2—H2B	108.6	C12—C11—C10	119.9 (3)
C3—C2—H2B	108.6	C13—C12—C11	121.2 (3)
H2A—C2—H2B	107.6	C13—C12—H12	119.4
C4—C3—C7	111.0 (2)	C11—C12—H12	119.4
C4—C3—C2	106.0 (2)	C14—C13—C12	119.6 (3)
C7—C3—C2	111.0 (2)	C14—C13—H13	120.2
C4—C3—C8	109.5 (2)	C12—C13—H13	120.2
C7—C3—C8	109.9 (2)	C15—C14—C13	120.1 (3)
C2—C3—C8	109.4 (2)	C15—C14—H14	119.9
C5—C4—C3	115.2 (2)	C13—C14—H14	119.9
C5—C4—H4A	108.5	C14—C15—C16	120.2 (3)
C3—C4—H4A	108.5	C14—C15—H15	119.9
C5—C4—H4B	108.5	C16—C15—H15	119.9
C3—C4—H4B	108.5	C11—C16—C15	120.4 (3)
H4A—C4—H4B	107.5	C11—C16—H16	119.8
O1—C5—C6	121.5 (3)	C15—C16—H16	119.8
O1—C5—C4	117.6 (2)	C18—C17—C22	120.1 (2)
C6—C5—C4	120.9 (2)	C18—C17—N1	121.1 (2)
C1—C6—C9	124.3 (2)	C22—C17—N1	118.6 (2)
C1—C6—C5	116.5 (2)	C17—C18—C19	120.0 (3)
C9—C6—C5	119.2 (2)	C17—C18—H18	120.0
C3—C7—H7A	109.5	C19—C18—H18	120.0
C3—C7—H7B	109.5	C18—C19—C20	119.4 (3)
H7A—C7—H7B	109.5	C18—C19—H19	120.3
C3—C7—H7C	109.5	C20—C19—H19	120.3
H7A—C7—H7C	109.5	C21—C20—C19	120.9 (3)
H7B—C7—H7C	109.5	C21—C20—Br1	120.2 (2)
C3—C8—H8A	109.5	C19—C20—Br1	118.9 (2)
C3—C8—H8B	109.5	C20—C21—C22	119.4 (3)
H8A—C8—H8B	109.5	C20—C21—H21	120.3
C3—C8—H8C	109.5	C22—C21—H21	120.3
H8A—C8—H8C	109.5	C21—C22—C17	120.1 (3)
H8B—C8—H8C	109.5	C21—C22—H22	120.0
C6—C9—S2	125.9 (2)	C17—C22—H22	120.0
			
C17—N1—C1—C6	172.3 (3)	C10—S1—C9—S2	4.9 (2)
C17—N1—C1—C2	−8.4 (4)	C9—S1—C10—C11	174.14 (19)
N1—C1—C2—C3	−152.6 (2)	S1—C10—C11—C16	109.1 (3)
C6—C1—C2—C3	26.7 (4)	S1—C10—C11—C12	−69.9 (3)
C1—C2—C3—C4	−53.7 (3)	C16—C11—C12—C13	1.0 (4)
C1—C2—C3—C7	66.9 (3)	C10—C11—C12—C13	−179.9 (3)
C1—C2—C3—C8	−171.7 (2)	C11—C12—C13—C14	−0.5 (5)
C7—C3—C4—C5	−68.6 (3)	C12—C13—C14—C15	−0.2 (5)
C2—C3—C4—C5	52.0 (3)	C13—C14—C15—C16	0.3 (5)
C8—C3—C4—C5	169.9 (2)	C12—C11—C16—C15	−0.8 (4)
C3—C4—C5—O1	158.1 (3)	C10—C11—C16—C15	−179.9 (3)
C3—C4—C5—C6	−23.4 (4)	C14—C15—C16—C11	0.2 (5)
N1—C1—C6—C9	3.5 (4)	C1—N1—C17—C18	−58.6 (4)
C2—C1—C6—C9	−175.8 (2)	C1—N1—C17—C22	125.4 (3)
N1—C1—C6—C5	−174.7 (2)	C22—C17—C18—C19	0.2 (4)
C2—C1—C6—C5	6.0 (4)	N1—C17—C18—C19	−175.6 (2)
O1—C5—C6—C1	170.7 (3)	C17—C18—C19—C20	0.8 (4)
C4—C5—C6—C1	−7.8 (4)	C18—C19—C20—C21	−1.1 (4)
O1—C5—C6—C9	−7.6 (4)	C18—C19—C20—Br1	176.4 (2)
C4—C5—C6—C9	173.9 (2)	C19—C20—C21—C22	0.3 (4)
C1—C6—C9—S2	3.1 (4)	Br1—C20—C21—C22	−177.1 (2)
C5—C6—C9—S2	−178.7 (2)	C20—C21—C22—C17	0.7 (4)
C1—C6—C9—S1	−179.3 (2)	C18—C17—C22—C21	−1.0 (4)
C5—C6—C9—S1	−1.2 (3)	N1—C17—C22—C21	174.9 (2)
C10—S1—C9—C6	−172.8 (2)		

### Hydrogen-bond geometry (Å, °)

**Table:**

*D*—H···*A*	*D*—H	H···*A*	*D*···*A*	*D*—H···*A*
N1—H1···S2	0.88 (1)	2.10 (2)	2.905 (2)	151 (3)
